# Co-Inoculating *Burkholderia vietnamiensis* B418 and *Trichoderma harzianum* T11W Reduced *Meloidogyne incognita* Infestation of Tomato Plants

**DOI:** 10.3390/microorganisms13061337

**Published:** 2025-06-09

**Authors:** Yanqing Jiang, Wenzhe Li, Jishun Li, Jindong Hu, Yanli Wei, Yilian Wang, Hetong Yang, Yi Zhou, Yuanzheng Wu, Shanshan Zhang

**Affiliations:** 1 Shandong Provincial Key Laboratory of Applied Microbiology, Ecology Institute, Qilu University of Technology (Shandong Academy of Sciences), Jinan 250103, China; yanqing597976@163.com (Y.J.); lwenzhe1008@163.com (W.L.); yewu2@sdas.org (J.L.); hujd@sdas.org (J.H.); yanli_wei@163.com (Y.W.); yilianwang@163.com (Y.W.); yanght@sdas.org (H.Y.); 2 School of Bioengineering, Qilu University of Technology (Shandong Academy of Sciences), Jinan 250353, China; 3 China—Australia Joint Laboratory for Soil Ecological Health and Remediation, Ecology Institute, Qilu University of Technology (Shandong Academy of Sciences), Jinan 250103, China; yi.zhou@adelaide.edu.au; 4 School of Agriculture, Food and Wine, The University of Adelaide, Adelaide 5064, Australia

**Keywords:** metagenomic analysis, *Burkholderia vietnamiensis*, *Trichoderma harzianum*, co-culture, root-knot nematodes, rhizosphere microbiome

## Abstract

Root-knot nematodes (RKNs; *Meloidogyne incognita*) pose a significant threat to tomato crops, necessitating sustainable control methods. This study investigated the inoculation efficacy of co-cultured *Burkholderia vietnamiensis* B418 and *Trichoderma harzianum* T11W compared with single-strain treatments for RKNs suppression and their influence on the structure and function of the rhizosphere microbiome. Co-inoculation with B418 + T11W achieved a 71.42% reduction in the disease index, significantly outperforming single inoculations of B418 (54.46%) and T11W (58.93%). Co-inoculation also increased plant height by 38.51% and fresh weight by 76.02% compared to the RKNs infested plants control, promoting robust tomato growth. Metagenomic analysis reveals that co-inoculation enhanced bacterial diversity, with 378 unique bacterial species and a high Shannon index, while fungal diversity decreased with *Trichoderma* dominance (83.31% abundance). Actinomycetota (46.42%) and Ascomycota (97.92%) were enriched in the co-inoculated rhizosphere, showing negative correlations with RKNs severity. Functional analysis indicates enriched metabolic pathways, including streptomycin and unsaturated fatty acid biosynthesis, enhancing microbial antagonism. Single inoculations altered pathways like steroid degradation (B418) and terpenoid biosynthesis (T11W), but co-inoculation uniquely optimized the rhizosphere microenvironment. These findings highlight co-inoculation with B418 + T11W effectively suppressing RKNs and fostering plant health by reshaping microbial communities and functions, offering a promising approach for sustainable agriculture.

## 1. Introduction

Root-knot nematodes (RKNs; *Meloidogyne incognita*) are widespread plant parasites that severely impact economically important crops [1]. Their strong parasitism and broad distribution make them a major threat to agriculture [2]. Traditionally, chemical control, along with agricultural practices and resistant varieties, has been used, but it has limitations regarding environmental and human health issues. With advances in science and growing environmental concern, biological control has gained significant attention [3]. The use of antagonistic plant growth-promoting rhizobacteria (PGPR), such as *Bacillus*, *Burkholderia*, *Pseudomonas*, and *Pasteuria*, has been reported to effectively control RKNs while promoting plant growth and yield [4,5]. Kim et al. found that the cell-free filtrate of *Burkholderia* sp. JB-2 exhibited strong nematicidal activity, achieving an 87.5% mortality rate against second-instar larvae (J2) within 2 days [6]. Liu et al. reported that the individual application of *B. vietnamiensis* B418 presented a high control efficiency of 71.15% against RKNs on watermelon in a field experiment, and the control efficiencies remained at 62.71% and 67.87% when combined with fosthiazate and avermectin, exhibiting slight incompatibility, which was still notably higher than when the nematicides were applied separately [7].

Microbial co-culture systems exhibit high-efficiency potentials in suppressing RKNs and plant pathogens by activating complex interspecies interactions [8]. Studies show that the synergistic effects of multiple beneficial microorganisms significantly enhance nematicidal efficacy. For example, after co-culturing *Bacillus amyloliquefaciens* Sneb709 and *Sinorhizobium fredii* Sneb183, the inhibition rate of the fermentation broth against J2 of *M. incognita* significantly increased to 84.17%, far exceeding the effect of single strains [9]. Siddiqui et al. demonstrated that culture filtrate extracts of *Trichoderma harzianum* Th6 and *Pseudomonas fluorescens* CHA0 enhanced nematicidal activity and reduced RKNs population density in tomato plants [10]. The application of microbial agents or stimulants has been reported to directly inhibit RKNs, trigger plant resistance, and enhance the biodiversity of antagonistic microbial communities [11]. The effect of co-culture depends on the specific strains involved and their relative proportions. Compared with mono-cultures, co-culturing not only influences the microbial growth but also has a greater effect on metabolism. There was no previous report regarding *Trichoderma* and *Burkholderia* co-culture for RKNs inhibition.

Microbiomes play a crucial role in soil and agro-ecosystems [12]. Soil metagenomics enables the exploration of diverse microbial communities without the need for cultivation [13,14]. Whole-genome sequencing has been widely used to study soil samples, uncovering the functional diversity of microbial metagenomes [15]. Rhizosphere microbiota support tomato plant growth and development, enhancing its overall health [16,17]. Studies show that co-cultures significantly increased microbial diversity and biomass in the rhizosphere while also isolating rare and novel bacterial species [18,19]. Li et al. found that RKNs parasitism significantly influenced the diversity and assembly of plant-associated microbial communities [20]. Similarly, Lu et al. reported a strong correlation between rhizosphere bacterial distribution in tobacco and RKNs infection, identifying 15 genera positively correlated and 42 genera negatively correlated with RKNs density [21]. Notably, some negatively correlated genera, such as *Pseudomonas* and *Bacillus*, are known to help suppress soil-borne pathogens. The combined use of two biocontrol strains significantly altered the composition of the soil bacterial community and influenced the relative abundance of beneficial microorganisms in the rhizosphere [22]. Kamalanathan et al. found that treatment with *Bacillus velezensis* VB7 and *T. koningiopsis* TK modified the fungal community structure in the rhizosphere, promoted the accumulation of diverse nematode-resistant biomolecules, induced defense mechanisms that inhibited nematode infection, and enhanced plant growth [23].

Previous studies have demonstrated that both *Burkholderia vietnamiensis* B418 and *Trichoderma harzianum* T11W are effective against RKNs [7,24,25]. This study aims to evaluate the effectiveness of a co-cultured fermentation broth of these two strains in controlling tomato RKNs compared to single-train treatments and analyze differences in the rhizosphere microbiome structure and function under single and co-inoculation conditions.

## 2. Materials and Methods

### 2.1. Strains and Media

The biocontrol strains *B. vietnamiensis* B418 (China General Microbiological Culture Collection Center, CGMCC No. 1212) and *T. harzianum* T11W (CGMCC No. 7938) were isolated and preserved by the Environmental Microbiology Laboratory, Ecology Institute of Shandong Academy of Sciences [7,24,25]. Both strains were preserved at −80 °C prior to use. *Meloidogyne incognita* was obtained from the roots of diseased tomato plants collected from vegetable-growing greenhouses, as previously described [7,24]. The tomato variety used in this study was Sweet Treasure, a nematode susceptible cultivar harvested from greenhouses in Zhangqiu District, Jinan City, Shandong Province.

Various culture media were prepared for microbial growth and experimentation. A tryptone yeast extract (TY) medium was prepared with peptone (10 g), yeast powder (1 g), CaCl_2_ (0.2 g), and distilled water (1 L), adjusted to a pH of 7.2–7.4. The potato dextrose agar (PDA) consisted of peeled potato (200 g), dextrose (20 g), and agar (15 g) dissolved in 1 L of distilled water. The potato dextrose broth (PDB) was similarly composed of peeled potato (200 g) and dextrose (20 g) in 1 L of distilled water. Additionally, Modified King’s B broth (MKB) contained casein amino acids (20 g L^−1^), glycerol (10 mL), K_2_HPO_4_ (1.5 g L^−1^), MgSO_4_·7H_2_O (1.5 g L^−1^), and distilled water (1 L), adjusted to pH 7.2 ± 0.2.

### 2.2. Co-Culture Conditions

For the preparation of *B. vietnamiensis* B418, bacterial stocks preserved at −80 °C were retrieved and inoculated onto TY plates. After single colonies developed, an inoculating loop was used to transfer the colonies into a TY liquid medium. The culture was incubated at 28 °C with shaking at 180 rpm for 12 h. The B418 bacterial suspension was diluted 10^6^ folds and spread onto TY plates, and then the individual colonies were counted to make the diluted suspension of 1 × 10^8^ CFU/mL for further experiments.

For *T. harzianum* T11W, fungal plugs stored in glycerol at −80 °C were inoculated onto PDA plates and incubated at 28 °C for 5 days. After incubation, spores were washed from the plates, and their concentration was determined using a hemocytometer. The spore suspension was then diluted to 1 × 10^8^ CFU/mL for use in co-culture experiments.

To establish the *B. vietnamiensis* B418 and *T. harzianum* T11W co-culture, 200 μL of B418 bacterial and T11W fungal spore suspensions were inoculated into 200 mL of MKB at a ratio of 1:1 in 1000 mL conical flasks. For the mono-culture, T11W and B418 were inoculated into 200 mL of MKB at the same inoculation ratio and cultivated in the same conditions, respectively. The cultures were incubated at 28 °C with shaking at 180 rpm for 14 days, resulting in the production of a mono- and co-culture fermentation broth.

### 2.3. Preparation of Root-Knot Nematodes (RKNs)

Tomato roots infected with RKNs were cut into small pieces and washed in the laboratory. The root fragments were soaked in a 0.5% NaClO solution for 5 min, then rinsed thoroughly by passing the suspension through 50 μm and 100 μm sieves under running water. The suspension was centrifuged at 3000 rpm for 3 min, after which the supernatant was discarded. An equal volume of 75% sucrose was added to the remaining suspension, followed by another centrifugation at 3000 rpm for 3 min to remove impurities.

The nematode suspension was further disinfected with a 0.1% NaClO solution for 3 min and then rinsed with sterile water under aseptic conditions. The nematode inoculum level was determined using an inverted microscope and adjusted to 200 eggs/mL, as previously described by Zuhair et al. [26].

### 2.4. Pot Experiment

Three-week-old tomato plants with uniform growth were transplanted into pots filled with RKNs-free soil, which was pre-tested by sucrose density centrifugation and microscopy examination to confirm the absence of nematodes. The RKNs-free soil has a pH value of 7.0, the organic matter content is 3%, and the soil texture is loam. After the tomato plants had been allowed to recover from transplantation for 7 days, select plants with uniform plant height for the experiment. The experimental design is summarized in Table 1, including four treatments (designated as RKNs, B418, T11W, and B418 + T11W). For each treatment, 20 mL of nematode suspension (200 eggs/mL) were infested into the pot, while 10 mL of 14-day fermentation broth of mono and co-culture were inoculated to tomato plants for B418, T11W, and B418 + T11W treatments, respectively. There were 16 replicates planted in 4 pots for each treatment, and they were randomly placed during the experiment. The mean temperature in the greenhouse was set to 28–35 °C during the day and 10–15 °C during the night, with 80–90% relative humidity. After 60 days of growth, plant height, fresh weight, and disease severity were measured at harvest. The disease index of the root knots (gall index) was assessed using the six-grade classification method [27] and calculated according to the standard formula [28]:

(1) $$\mathrm{Disease}\;\mathrm{index}=\sum \frac{Number\;of\;plants\;of\;each\;disease\;grade\times Representative\;value\;of\;each\;disease\;grade}{Total\;number\;of\;plants\times representative\;value\;of\;the\;highest\;disease\;grade}\times 100$$

### 2.5. Metagenomic Sequencing and Bioinmatics

Three tomato plants out of four pots were randomly selected from each treatment. The bulk soil around the roots was shaken off, and any soil tightly attached to the root segment within the range of 0–4 mm was gently brushed off. The collected rhizosphere soil samples were stored at −80 °C for further analysis. DNA was extracted from soil samples using a Power Soil DNA Isolation Kit (Qiagen, Hilden, Germany). One microliter of RNase A was added to the tube to digest RNA, followed by incubation at 37 °C for 15 min. The concentration and purity of the extracted DNA were assessed using a Nanodrop spectrophotometer and agarose gel electrophoresis. Sequencing libraries were prepared using an NEBNext^®^ Ultra^TM^ DNA Library Prep Kit for Illumina (NEB, Ipswich, MA, USA), according to the manufacturer’s instructions, targeting a fragment size of 350 bp. The libraries were then sequenced on an Illumina NovaSeq platform, generating paired-end reads.

The raw sequencing data was preprocessed to improve quality by removing sequencing adapters and filtering out reads containing low-quality bases or sequences shorter than 50 bp using Kneaddata (Version 0.7.4) software [29]. Additionally, Bowtie2 (Version 2.3.5.1) software [30] was used to eliminate host plant genomic DNA by filtering out reads of the host origin before further analysis. Finally, FastQC (Version 0.11.9) was employed to assess the effectiveness and reliability of the quality control process. Kraken2 (Version 2.0.7-beta) [31], along with a microbial database containing sequences from bacteria, fungi, archaea, and viruses (screened from the NT nucleic acid database and the RefSeq (https://www.ncbi.nlm.nih.gov/refseq/, accessed on 8 November 2023) whole genome database of NCBI (https://www.ncbi.nlm.nih.gov/); accessed on 8 November 2023, was used to identify the species present in the samples. Bracken (Version 2.0) [32] was then employed to estimate the relative abundance of these species. Kraken2, a k-mer-based classification tool, utilized a local database comprising 16,799 known bacterial genomes. Following quality control and host sequence removal, clean reads were aligned to the UniRef90 database (http://www.uniprot.org/uniref/; accessed on 8 November 2023) using HUMAnN3 (Version 3.6) software [33], which operates with DIAMOND (Version 0.8.22) for sequence alignment. Based on the correspondence between UniRef90 IDs and the KEGG database (https://www.kegg.jp/; accessed on 8 November 2023), functional annotation data and relative abundance tables were generated.

### 2.6. Statistical Analysis

The data were statistically analyzed using SPSS 26.0 (SPSS, Chicago, IL, USA) and PRISM 8.3.0.538 software (GraphPad Software, San Diego, CA, USA). Analysis of variance (ANOVA) was performed following Tukey’s honest significant difference (HSD) test to identify significant differences among the samples (*p* < 0.05). LEfSe (Linear Discriminant Analysis Effect Size) was used to identify rhizosphere microbiome functions that showed significant differences between treatments.

## 3. Results

**Figure 1:**
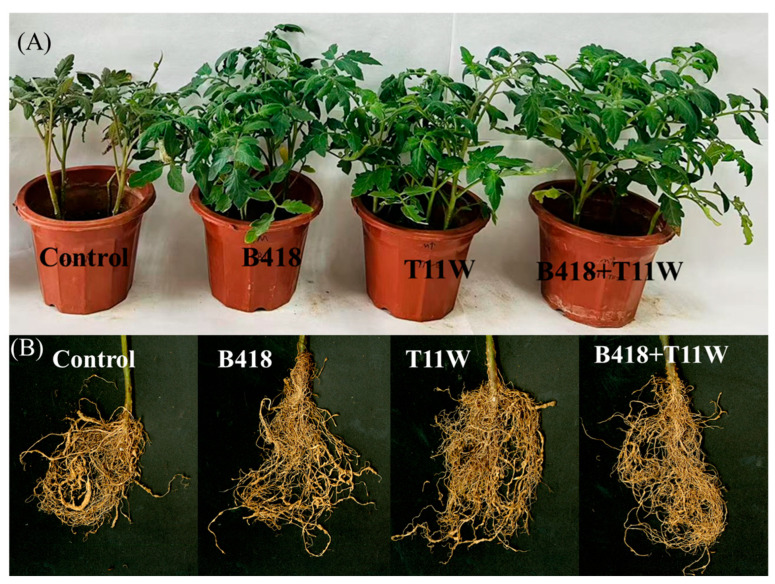
Results of tomato pot experiment in greenhouse conditions. (**A**) Potted plant images of different treatments; (**B**) root development of different treatments. Control: RKNs, B418: *B. vietnamiensis* B418 + RKNs, T11W: *T. harzianum* T11W + RKNs, B418 + T11W: *B. vietnamiensis* B418 + *T. harzianum* T11W + RKNs, RKNs: root-knot nematodes.

Tomato plants subjected to the RKNs treatment without microbial inoculation exhibited dwarfing and wilting symptoms 60 days after the addition of the nematode suspension (Figure 1). In contrast, plants treated with B418 + T11W and T11W alone grew taller than those treated with B418 alone, with average heights of 23.31–24.28 cm compared to 22.14 cm (Table 2). The B418 + T11W treatment resulted in a 38.51% increase in plant height relative to the RKNs control, while T11W and B418 treatments led to increases of 32.97% and 26.30%, respectively. There was no significant difference in fresh weight between the B418 + T11W and T11W treatments, both of which yielded the highest fresh weights among the treatments. The B418 + T11W treatment increased fresh weight by 76.02% compared to the RKNs control, while T11W and B418 increased it by 65.78% and 39.34%, respectively. Specifically, the combined treatment of B418 + T11W significantly reduced the disease index of tomato plants to 26.67 ± 1.56 compared with the individual treatments of B418 and T11W, which were 38.33 ± 1.56 and 42.50 ± 1.02, respectively (*p* < 0.05) (Table 2). The co-inoculation with B418 + T11W achieved a 71.42% reduction in the disease index compared to the RKNs control, significantly outperforming single inoculations of B418 (54.46%) and T11W (58.93%).

**Figure 2:**
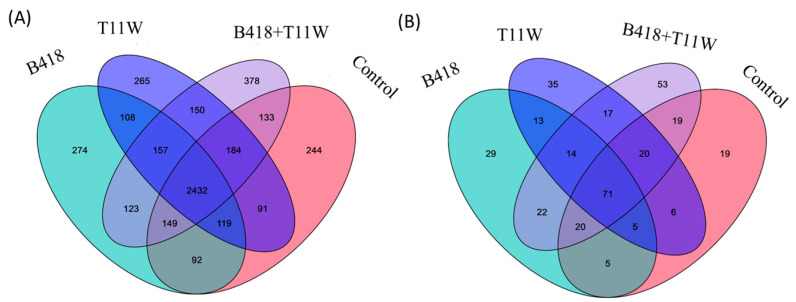
(**A**) Comparison of OTUs at bacterial species level in different treatments. (**B**) Comparison of OTUs at fungal species level in different treatments. Control = RKNs, B418 = *B. vietnamiensis* B418 + RKNs, T11W = *T. harzianum* T11W + RKNs, B418 + T11W = *B. vietnamiensis* B418 + *T. harzianum* T11W + RKNs.

A total of 2432 bacterial species were detected in the rhizosphere soil across all four treatments (Figure 2A). The majority of these species were common to all treatments. Notably, 378 species were uniquely found in the rhizosphere of plants inoculated with the B418 + T11W combination, which was higher than the number of unique species in the other treatments. A similar pattern was observed for fungi, where most species were shared across treatments, but the co-culture group also harbored the highest number of unique fungal species (Figure 2B).

**Figure 3:**
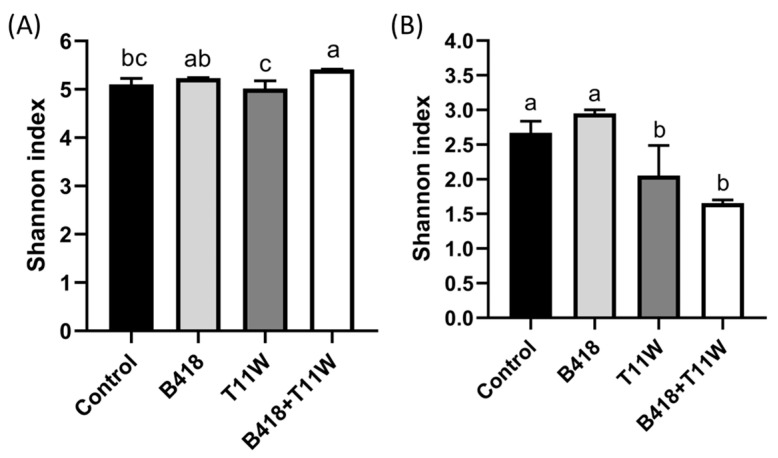
Shannon diversity index of (**A**) bacterial and (**B**) fungal communities in the tomato rhizosphere under different inoculation treatments. Control: RKNs; B418: *B. vietnamiensis* B418 + RKNs; T11W: *T. harzianum* T11W + RKNs; B418 + T11W: *B. vietnamiensis* B418 + *T. harzianum* T11W + RKNs. RKNs: root-knot nematodes. Error bar indicates standard error. The same letter indicates no significant difference based on Duncan’s test.

The rhizosphere bacterial community in the co-culture B418 + T11W treatment exhibited the highest Shannon diversity index, followed by the B418 treatment (Figure 3A). In contrast, the non-inoculated control and T11W treatments showed lower Shannon indices compared to the co-culture treatment. Conversely, for the fungal community, the Shannon index was lowest in the B418 + T11W treatment, while the control and B418 treatments displayed the highest fungal diversity (Figure 3B).

**Figure 4:**
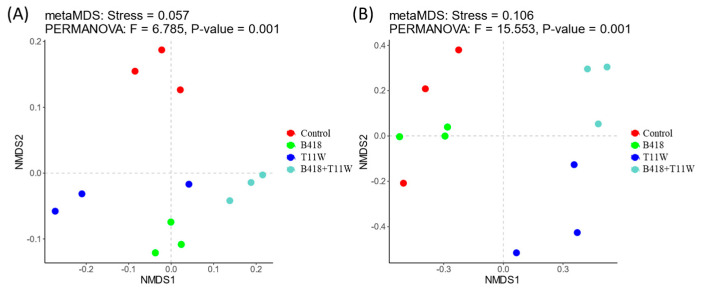
Non-metric multidimensional scaling based on the Bray–Curtis distance for the composition of the (**A**) bacterial species and (**B**) fungal species in the tomato rhizosphere under different inoculation treatments. Control: RKNs; B418: *B. vietnamiensis* B418 + RKNs; T11W: *T. harzianum* T11W + RKNs; B418 + T11W: *B. vietnamiensis* B418 + *T. harzianum* T11W + RKNs. RKNs: root-knot nematodes. (**A**) PERMANOVA’s pseudo-F = 6.785, *p*-value = 0.001; (**B**) PERMANOVA’s pseudo-F = 15.553, *p*-value = 0.001.

The microbial community composition in the rhizosphere soil varied among the four treatments (Figure 4). The influence of different inoculation treatments on the bacterial community resembled their effect on the fungal community. Specifically, the microbial community structure in the rhizosphere of plants inoculated with the B418 + T11W combination differed from that observed in plants treated with either strain individually.

**Figure 5:**
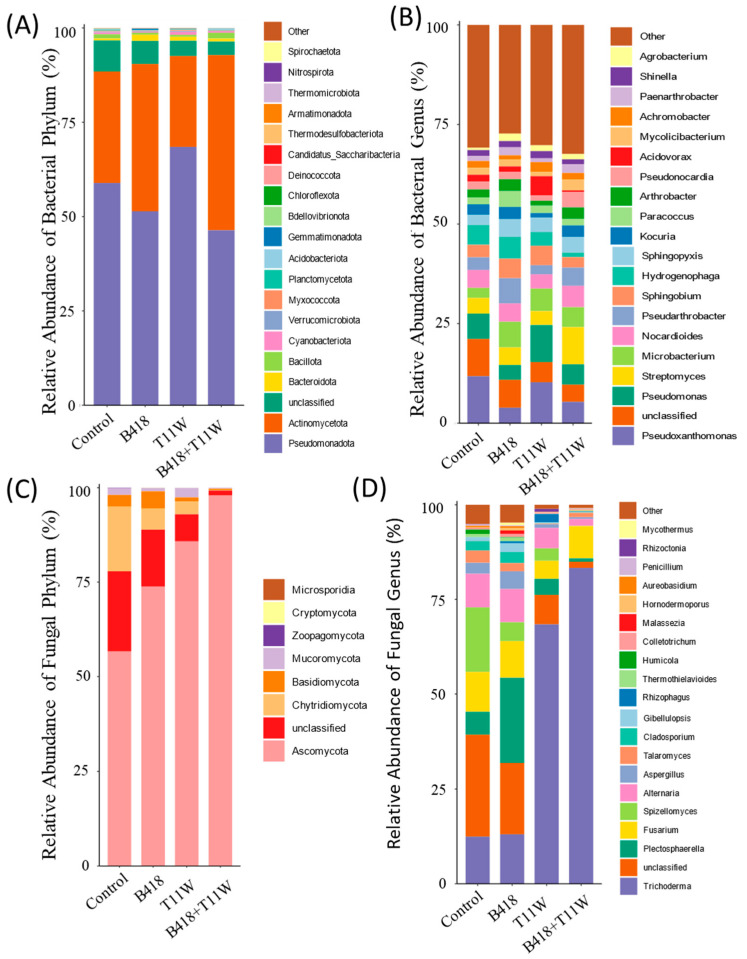
Relative abundances of microbial communities under different inoculation treatments. (**A**) Bacterial phylum; (**B**) bacterial genus; (**C**) fungal phylum; (**D**) fungal genus. Control: RKNs; B418: *B. vietnamiensis* B418 + RKNs; T11W: *T. harzianum* T11W + RKNs; B418 + T11W: *B. vietnamiensis* B418 + *T. harzianum* T11W + RKNs. RKNs: root-knot nematodes.

At the phylum level, Pseudomonadota and Actinomycetota were the dominant bacterial groups across all four treatments. In the control treatment, their relative abundances were 58.91% and 29.53%, respectively. In the B418-treated rhizosphere soil, Pseudomonadota accounted for 51.4% and Actinomycetota for 39.05%. For the T11W treatment, Pseudomonadota made up 68.48%, while Actinomycetota represented 24.09%. In the rhizosphere of plants treated with the B418 + T11W co-culture, Actinomycetota showed the highest relative abundance at 46.42%, closely followed by Pseudomonadota at 46.38% (Figure 5A). The predominance of Actinomycetota and Pseudomonadota in the rhizosphere under B418 + T11W, B418, and T11W treatments may be a key factor contributing to the suppression of RKNs.

The bacterial genera *Pseudoxanthomonas* and *Pseudomonas* were dominant across all rhizosphere soil samples. *Pseudoxanthomonas* had the highest relative abundance in the control group, followed by the T11W treatment, while its abundance decreased in the B418 + T11W co-culture treatment. In contrast, *Streptomyces* reached its highest relative abundance (9.35%) in the B418 + T11W treatment compared to 4.42% in the B418 group, 3.52% in T11W, and 3.94% in the control group (Figure 5B).

In terms of the fungal community, Ascomycota was the most dominant phylum across all treatments. Its relative abundance was 56.63% in the non-inoculated control, increasing to 73.83% in the B418 group and 85.78% in the T11W group. The highest abundance was observed in the B418 + T11W co-culture treatment, reaching 97.92%, indicating that Ascomycota may play a key role in suppressing RKNs and enhancing plant growth (Figure 5C).

At the genus level, *Trichoderma* was dominant in all treatments. The co-culture B418 + T11W treatment showed a substantial increase in *Trichoderma* abundance compared to the control, rising from 12.44% to 83.31%. In the B418 and T11W treatments, Trichoderma accounted for 13.05% and 68.38%, respectively. The B418 + T11W treatment also markedly reduced the abundance of unclassified fungi from 26.89% in the control to just 1.65%. *Plectosphaerella* was most abundant in the B418 group (22.38%), followed by the control (6.03%) and T11W (4.24%), with the lowest abundance in the B418 + T11W group (0.86%) (Figure 5D).

**Figure 6:**
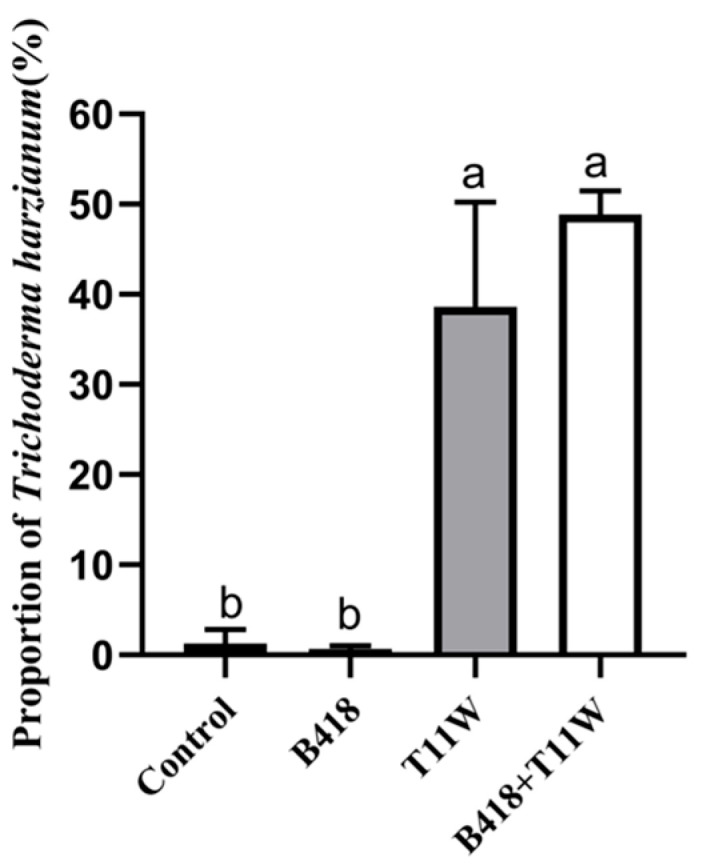
The proportions of *Trichoderma harzianum* under different inoculation treatments. Control: RKNs; B418: *B. vietnamiensis* B418 + RKNs; T11W: *T. harzianum* T11W + RKNs; B418 + T11W: *B. vietnamiensis* B418 + *T. harzianum* T11W + RKNs. RKNs: root-knot nematodes. Error bar indicates standard error. The same letter indicates no significant difference based on Duncan’s test.

The marked increase in *Trichoderma* levels in both the T11W and B418 + T11W treatments indicates that the inoculation significantly boosted its presence (Figure 6). The relative abundance of *T. harzianum* mirrored that of the *Trichoderma* genus, with the highest level found in the B418 + T11W group (48.89%), followed by the T11W group (38.63%). No significant difference in *T. harzianum* abundance was observed between the T11W and B418 + T11W treatments or between the control and B418 treatments.

**Figure 7:**
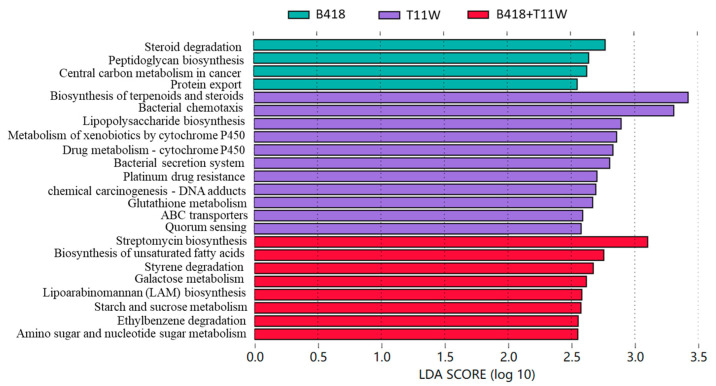
Functional pathways that showed significant differences from other treatments were identified using linear discriminant analysis effect size (LEfSe), with linear discriminant analysis (LDA) scores greater than 2.5 and a significance threshold of *p* < 0.05. Control: RKNs; B418: *B. vietnamiensis* B418 + RKNs; T11W: *T. harzianum* T11W + RKNs; B418 + T11W: *B. vietnamiensis* B418 + *T. harzianum* T11W + RKNs. RKNs: root-knot nematodes.

Functional pathways among different treatments were presented in Figure 7 using linear discriminant analysis effect size (LEfSe), with linear discriminant analysis (LDA) scores greater than 2.5 and a significance threshold of *p* < 0.05. In the B418 treatment group, four metabolic pathways showed significantly different abundances compared to the other treatments. These pathways included steroid degradation, peptidoglycan biosynthesis, central carbon metabolism in cancer, and protein export, corresponding to the KEGG functional categories of metabolism, human diseases, genetic information processing, and cellular processes, respectively. Among these, the key genes involved in the central carbon metabolism in the cancer pathway were G6PD and *zwf*, both encoding glucose-6-phosphate 1-dehydrogenase.

In the T11W treatment group, significantly enriched metabolic pathways were categorized into four major functional classes. The first was cellular processes, represented by quorum sensing and bacterial chemotaxis. Key genes identified in bacterial chemotaxis included *mcp* and *fliM*, encoding the methyl-accepting chemotaxis protein and the flagellar motor switch protein FliM, respectively. The second category, environmental information processing, included the ABC transporter system and bacterial secretion system. The key genes within the ABC transporters were *phnE*, *livK*, and *urtE*, which encode proteins associated with phosphonate transport, branched-chain amino acid transport, and urea transport, respectively. The third category, human diseases, involved pathways related to chemical carcinogenesis−DNA adducts and platinum drug resistance− both associated with the genes GST and *gst*, which encode glutathione S-transferase. The fourth category, metabolism, comprised pathways including glutathione metabolism, drug metabolism (cytochrome P450), xenobiotic metabolism (cytochrome P450), lipopolysaccharide biosynthesis, and biosynthesis of terpenoids and steroids. In these pathways, GST and *gst* again appeared as key genes involved in glutathione S-transferase activity.

In the B418 + T11W co-inoculation group, all significantly enriched pathways were related to metabolic functions. These included amino sugar and nucleotide sugar metabolism, ethylbenzene degradation, starch and sucrose metabolism, lipoarabinomannan (LAM) biosynthesis, galactose metabolism, styrene degradation, biosynthesis of unsaturated fatty acids, and streptomycin biosynthesis. The gene *glk*, encoding glucokinase, was identified as a key gene in both amino sugar and nucleotide sugar metabolism, as well as in starch and sucrose metabolism. Additionally, *bglB* (encoding beta-glucosidase) was involved in starch and sucrose metabolism. For ethylbenzene degradation, the key genes fadA and fadI encoding acetyl-CoA acyltransferase were identified. In galactose metabolism, the genes *glk*, *pfkA*, and PFK (encoding glucokinase and 6-phosphofructokinase) were prominent. Finally, for streptomycin biosynthesis, the key genes GCK and INO1, encoding glucokinase and myo-inositol-1-phosphate synthase, respectively, were significantly enriched.

## 4. Discussion

These findings demonstrate that co-inoculation with B418 and T11W provided the most effective control of root-knot nematodes (RKNs) in tomato plants, achieving a control efficacy of 71.43%. Individually, *T*. *harzianum* T11W and *B*. *vietnamiensis* B418 also showed moderate suppression of RKNs, with control rates ranging from 54% to 58%. However, the combined inoculation of both strains resulted in a significantly enhanced control effect. This co-inoculation strategy not only suppressed nematode infestation more effectively but also promoted tomato plant growth. Similarly, Sharma et al. reported that co-inoculation with *Rhizophagus irregularis* and *Pseudomonas* enhanced tomato growth and reduced nematode disease compared to single-strain treatments [34], although the inoculated beneficial microbes species differs from the present study. Notably, the present study further elucidates the mechanisms behind the enhanced performance of co-inoculation, revealing that it modulates both the composition and function of the rhizosphere microbiome.

Significant shifts in both bacterial and fungal community compositions were observed in the rhizosphere soil of tomato plants subjected to different inoculation treatments, with potential associations identified between these microbial changes and the incidence of RKNs disease. At the phylum level in the bacterial community, the relative abundance of Actinomycetota notably increased following microbial inoculation, with the most pronounced rise observed in the B418 + T11W co-inoculation treatment. This increase may play a crucial role in enhancing the suppression of tomato RKNs, as previous research has highlighted the nematode-inhibitory properties of Actinomycetota [35,36]. Moreover, actinomycetes are commonly utilized in agriculture as biocontrol agents and growth-promoting microbes for vegetable crops [37,38]. In the fungal community, the abundance of Ascomycota showed a negative correlation with RKNs disease severity in tomato. This observation aligns with the findings by Kamalanathan et al., who reported the highest levels of Ascomycota in the rhizosphere of tomato plants treated with biocontrol agents [23].

A significant increase in the proportion of *Trichoderma* spp. was observed in the rhizosphere microbial community of tomato plants under the T11W and B418 + T11W treatments compared to the control and B418 treatments. This increase is likely due to the introduction of the T11W strain in both groups. The dominance and persistence of *Trichoderma* spp. in the rhizosphere following inoculation align with the findings from previous studies. For instance, Wu et al. reported that *Trichoderma* spp. had a significantly higher relative abundance in treatments with a compound *Trichoderma* agent (CTA) than in water controls or compound fertilizer treatments, and it represented the most dominant genus in the CTA group [39].

Zhang et al. [40] demonstrated that treatment with *Trichoderma* biofertilizer significantly altered the fungal community structure in grassland rhizosphere soil, notably increasing the abundance of *Archaeorhizomyces* and *Trichoderma* while reducing the presence of *Ophiosphaerella*. Similarly, Halifu et al. [41] reported that *Trichoderma* spp. was the dominant fungal genus in the rhizosphere of *Pinus sylvestris var. mongolica* seedlings following inoculation with *T. harzianum* E15 and *T. virens* ZT05. The persistent dominance of *Trichoderma* spp. in rhizosphere soils has been widely attributed to their rapid growth rate, strong vitality, ability to quickly colonize available space, and efficient nutrient uptake. In contrast, *Burkholderia* spp. was not identified as a dominant genus in the bacterial communities of the B418 and B418 + T11W treatments, likely due to the higher overall bacterial diversity present in the rhizosphere soil under these conditions.

In this study, the application of microbial inoculants led to an increase in soil bacterial diversity, with the co-inoculation of B418 + T11W resulting in the most pronounced enhancement. Similar findings were reported by Zheng et al., where the introduction of *Pantoea jilinensis* D25 significantly elevated bacterial diversity in the tomato rhizosphere, particularly among beneficial taxa [42]. Likewise, Jie et al. observed that inoculation with *Rhizophagus intraradices* enhanced both bacterial and fungal diversity in the rhizosphere soil of soybean [43]. Soil microbial diversity plays a crucial role in determining soil health and is essential for sustainable soil development [44,45]. A decline in microbial diversity can impair soil ecosystem functions and productivity, as the richness and composition of microbial communities are fundamental to ecological processes [46]. High microbial diversity is also associated with improved plant protection against soil-borne pathogens [47]. The observed shift in microbial community composition following biological inoculation may, therefore, contribute to the suppression of root-knot nematode disease. On the other hand, the diversity of fungal communities in the rhizosphere decreased following inoculation with T11W or the B418 + T11W co-culture. This reduction is likely due to the dominance of *Trichoderma* introduced by these treatments, which may have outcompeted and limited the presence of other fungal species. There were usually plant pathogens combined with RKNs infestation due to the host vulnerability. The dominance of *Trichoderma* spp. in rhizosphere soils could, therefore, not only inhibit RKNs infestation but also prevent potential soil-borne diseases caused by secondary pathogens.

The LDA of functional pathway differences across treatment groups reveals that each inoculant exerted distinct influences on the functional dynamics of the tomato rhizosphere microbiome. In the B418 treatment group, rhizosphere microbes and enzymes associated with steroid degradation were particularly active, potentially contributing to the breakdown and transformation of steroid compounds in the soil environment [48]. Additionally, the upregulation of peptidoglycan biosynthesis in this group is likely linked to key bacterial physiological processes, particularly the formation of cell walls [49].

For the T11W treatment, notable enrichment was observed in pathways related to terpenoid and steroid biosynthesis, as well as bacterial chemotaxis. This suggests that the inoculation may stimulate the microbial production of terpenoids and steroids, compounds known to play roles in microbial ecology and physiological regulation [50]. Furthermore, the enhanced bacterial chemotaxis could improve microbial mobility, aiding in nutrient acquisition and the evasion of environmental stressors [51].

In the co-inoculated B418 + T11W treatment, there was a marked increase in metabolic activity related to the biosynthesis of streptomycin and unsaturated fatty acids. Streptomycin production can influence microbial antagonism and contribute to beneficial plant–microbe interactions, which are key for promoting plant health and improving soil fertility [52]. Meanwhile, the elevated synthesis of unsaturated fatty acids may impact microbial cell membrane fluidity and integrity, thereby influencing microbial metabolism and viability [53].

## 5. Conclusions

In summary, the use of biocontrol agents *B. vietnamiensis* B418, *T. harzianum* T11W, and their combination (B418 + T11W) effectively suppressed tomato RKNs, with the co-inoculation treatment showing the most pronounced control effect, reaching 71.42% reduction in the disease index. Additionally, these treatments significantly altered the composition and function of the rhizosphere microbial community. Application of the biocontrol agents notably enhanced soil bacterial diversity, with the most substantial increase observed in the B418 + T11W treatment. At the bacterial phylum level, Actinomycetota abundance increased markedly following biocontrol application and showed a strong negative correlation with RKNs incidence. For fungi, Ascomycota abundance also rose significantly under biocontrol treatments and was similarly negatively correlated with RKNs disease severity. Overall, the introduction of microbial inoculants reshaped the rhizosphere microbial structure, boosting the presence of beneficial microorganisms. The underlying mechanism involves the modulation of microbial metabolic pathways, which not only suppressed RKNs but also improved the soil microenvironment, thereby supporting healthier and more vigorous tomato plant growth.

## Figures and Tables

**Table 1:** Experimental designs for pot experiment.

No.	Name	Description
1	RKNs	20 mL nematode suspension (200 eggs/mL)
2	B418	20 mL nematode suspension (200 eggs/mL), 10 mL 14-day B418 fermentation broth (fermentation broth with bacteria cells)
3	T11W	20 mL nematode suspension (200 eggs/mL), 10 mL 14-day T11W fermentation broth (fermentation broth with mycelia cells)
4	B418 + T11W	20 mL nematode suspension (200 eggs/mL), 10 mL 14-day B418 + T11W fermentation broth (fermentation broth with bacteria and mycelia cells)

**Table 2:** The plant height, fresh weight, disease index, and control effect of tomato in the different treatments 60 days after planting.

Treatments	Plant Height (cm)	Fresh Weight (g)	Disease Index
RKNs	17.53 ± 0.54 c	4.88 ± 0.24 c	93.33 ± 1.56 a
B418	22.14 ± 0.59 b	6.80 ± 0.37 b	38.33 ± 1.56 b
T11W	23.31 ± 0.58 ab	8.09 ± 0.19 a	42.50 ± 1.02 b
B418 + T11W	24.28 ± 0.52 a	8.59 ± 0.26 a	26.67 ± 1.56 c

B418 = *B. vietnamiensis* B418 + RKNs, T11W = *T. harzianum* T11W + RKNs, B418 + T11W = *B. vietnamiensis* B418 + *T. harzianum* T11W + RKNs. Error bar indicates standard error. The same letter indicates no significant difference based on Duncan’s test.

## Data Availability

The data that support the findings of this study are available from the corresponding authors upon reasonable request.
